# Association of medication non-adherence with short-term allograft loss after the treatment of severe acute kidney transplant rejection

**DOI:** 10.1186/s12882-019-1563-z

**Published:** 2019-10-17

**Authors:** Ahmed Al-Sheyyab, Laura Binari, Mohammed Shwetar, Everly Ramos, Meghan E. Kapp, Stefanie Bala, Nikita Wilson, Rachel C. Forbes, J. Harold Helderman, Khaled Abdel-Kader, Beatrice P. Concepcion

**Affiliations:** 1 Department of Medicine, Division of Nephrology and Hypertension, Vanderbilt University Medical Center, 1161 21st Ave. S., MCN S-3223, Nashville, TN 37232 USA; 2 School of Medicine, Vanderbilt University, Nashville, TN USA; 3 Department of Pathology, Microbiology and Immunology, Vanderbilt University Medical Center, Nashville, TN USA; 4 Department of Pharmacy, Vanderbilt University Medical Center, Nashville, TN USA; 5 Department of Surgery, Division of Kidney and Pancreas Transplantation, Vanderbilt University Medical Center, Nashville, TN USA

**Keywords:** Non-adherence, Kidney transplantation, Graft loss, Acute rejection

## Abstract

**Background:**

Medication non-adherence is a risk factor for acute kidney transplant rejection. The association of non-adherence with short-term allograft loss in patients who develop acute rejection and are subsequently treated with maximal therapy is unknown.

**Methods:**

We conducted a retrospective single center cohort study of adult patients who developed acute rejection from January 2003 to December 2017 and were treated with lymphocyte depletion. Clinicopathologic characteristics including adherence status were collected and descriptive statistics utilized to compare groups. The primary outcome was all-cause graft loss at 6 months after acute rejection treatment. A multivariable logistic regression quantified the association of non-adherence with the outcome.

**Results:**

A total of 182 patients were included in the cohort, of whom 71 (39%) were non-adherent. Compared to adherent patients, non-adherent patients were younger (mean age 37y vs 42y), more likely to be female (51% vs 35%) and developed acute rejection later (median 2.3y vs 0.5y from transplant). There were no differences in estimated glomerular filtration rate or need for dialysis on presentation, Banff grade, or presence of antibody mediated rejection between the 2 groups. Overall, 48 (26%) patients lost their grafts at 6 months after acute rejection treatment. In adjusted analysis, non-adherence was associated with all-cause graft loss at 6 months after acute rejection treatment [OR 2.64 (95% CI 1.23–5.65, *p* = 0.012].

**Conclusions:**

After adjusting for common confounders, non-adherent patients were at increased risk for short-term allograft loss after a severe acute rejection despite lymphocyte depletion. This finding may aid clinicians in risk stratifying patients for poor short-term outcomes and treatment futility.

## Background

Acute kidney transplant rejection is a major cause of allograft loss. Treatment of acute rejection is usually determined by the clinical scenario and pathologic findings on biopsy. The presence of acute vascular rejection (Banff grade IIA, IIB, or III, or Cooperative Clinical Trials in Transplantation [CCTT] Types II or III) [1, 2] signifies a severe type of acute rejection and is traditionally treated with high dose intravenous steroids and a T-lymphocyte depleting agent [3, 4]. Additionally, acute cellular rejection that does not respond to high dose steroids may warrant escalation to T-lymphocyte depleting therapy [5]. Treatment of acute rejection with T-lymphocyte depleting agents is not without risks. Adverse effects include cytokine release syndrome, serum sickness, thrombocytopenia, lymphopenia, fever and an increased risk of infections and malignancy [6–9]. Although anti-thymocyte globulin (ATG), for example, has a relatively short half-life estimated to be between 2 and 3 days, lymphopenia often lasts for months and can last up to a year [6].

Clinical decision making to proceed with T-lymphocyte depleting therapy in a patient with severe acute rejection can at times be challenging. Identifying clinical predictors for short-term allograft loss despite aggressive acute rejection treatment may aid clinicians in risk stratifying patients for treatment futility and individualizing treatment decisions. For example, a clinician may decide to forego additional treatment and to focus instead on end-stage renal disease (ESRD) planning in a patient who is unlikely to respond to treatment or whose allograft is likely to fail within a few months despite T-lymphocyte depleting therapy.

Known clinical and pathologic factors for poor outcomes in acute rejection include black race, late onset of acute rejection, higher histological grade, and vascular involvement [10–15]. Although non-adherence is a known risk factor for acute rejection [16], its relationship with short-term allograft loss after treatment of an acute rejection episode is not well described. In this study we aimed to determine the association of medication non-adherence with allograft loss occurring within 6 months of acute rejection treatment with a T-lymphocyte depleting agent.

## Methods

### Study setting and participants

Inclusion criteria for this single center, retrospective cohort study were: age ≥ 18 years old, biopsy-proven acute rejection from January 2003 to December 2017, and receipt of maximal rejection therapy defined as treatment with a T-lymphocyte depleting agent, with or without antibody-mediated rejection (AMR) treatment. The rationale for having treatment with a T-lymphocyte depleting agent as an inclusion criteria was to have a cohort of patients that received the maximal therapy that our center would administer. The outcome (short-term graft loss) therefore could not be attributed to the withholding of additional treatment that could potentially impact graft survival. T-lymphocyte depletion consisted of treatment with any of the following: rabbit ATG (1.5 mg/kg) or horse ATG (10–15 mg/kg) for 7–14 days, alemtuzumab 30 mg for one dose, or muromonab-CD3 (OKT3) 5 mg daily for 10–14 days.

Recipients of dual solid organ transplants were excluded from the study. All study patients were followed at the Vanderbilt Kidney Transplant Clinic in Nashville, Tennessee as part of routine clinical care. Study follow-up ended at time of all-cause graft loss or at the end of the study (December 2018). The Vanderbilt University Medical Center Institutional Review Board (IRB#180289) approved the study.

### Adherence status

Patient adherence status (dichotomous) was adjudicated clinically at the time of patient presentation with corresponding documentation in the medical record. A retrospective review of clinical notes (initial history of present illness, progress notes and discharge summary) during the patient’s hospital stay for acute rejection was performed by at least one author. A patient was classified as being non-adherent if a clinical note stated that the patient was non-adherent to taking immunosuppressant medications. Patients with undetectable immunosuppressive levels were not necessarily classified as being non-adherent as there could be reasons other than non-adherence that lead to undetectable immunosuppressive levels.

### Covariates

Patient [i.e., age, gender, race/ethnicity, cause of ESRD, maintenance immunosuppression regimen, steroid withdrawal regimen, posttransplant nadir serum creatinine (SCr), recent baseline SCr, history of prior rejection, estimated glomerular filtration rate (eGFR) on presentation, dialysis dependence on presentation, undetectable immunosuppressive levels on presentation, presence of donor specific antibody (DSA), times to rejection from last follow-up and from transplant, lymphocyte-depleting agent used to treat the acute rejection episode], transplant (i.e., living versus deceased donor) and pathologic characteristics [i.e., Banff grade (I(A + B), II(A + B), or III), presence of AMR (presence of 2 out of 3 of the following: microcirculation inflammation, C4d positivity, and DSA positivity), percentages of interstitial fibrosis and glomerulosclerosis, presence of plasma cell or eosinophil-rich rejection, and transplant glomerulopathy] were collected via review of the electronic medical record and chart abstraction of biopsy reports.

### Outcomes

The primary outcome was all-cause graft loss within 6 months of acute rejection treatment. Graft loss was defined as return to chronic dialysis, re-transplantation or death. Secondary outcomes were all-cause graft loss within 12 months of acute rejection treatment and overall all-cause graft loss.

### Statistical analysis

Descriptive statistics were used to compare important clinical and pathological characteristics between groups. Categorical variables were described using frequencies and proportions and continuous variables were described using means (standard deviations) and medians (interquartile range). Two-sample t-test, Wilcoxon rank sum test and chi-square test were utilized as appropriate to compare groups.

A multivariable logistic regression model was used to examine the association of adherence status (primary predictor of interest) with all-cause graft loss within 6 (primary outcome) or 12 (secondary outcome) months of acute rejection treatment. The primary and secondary outcomes included patients who were dialysis-dependent on presentation. Based on literature review, clinical experience, and plausible linkages to the outcome of interest, the following variables were selected a priori for inclusion in the model: eGFR at presentation, Banff grade, presence of AMR, and degree of interstitial fibrosis (measured as the percentage of fibrosis in a given core specimen).

A Cox proportional hazards model was used to determine the association of adherence status with the risk of all-cause graft loss, after adjustment for the same covariates described above with the addition of age at rejection, race/ethnicity, type of transplant, nadir SCr and T-lymphocyte depleting agent used. The proportional-hazards assumption test based on Schoenfeld residuals was utilized to verify the proportionality assumption for the model. Due to the relatively high proportion of events, a sensitivity analysis was performed for the primary outcome using multivariable poisson regression with robust variance analysis [17]. A *p*-value < 0.05 was considered statistically significant. All analyses were performed using STATA SE version 15.0 (StataCorp, College Station, TX).

## Results

Between January 2003 and December 2017, we identified 433 cases of biopsy-proven acute rejection including 182 (42%) patients treated with a T-lymphocyte depleting agent who comprised the cohort. Median (IQR) follow-up time after the rejection episode was 21.5 (4.6–74.2) months. Non-adherence to medical therapy was common (*n* = 71, 39%). Patient, transplant and pathologic characteristics are shown in Table 1. Non-adherent patients were generally younger at the time of rejection, were more likely to be female and treated with a steroid withdrawal protocol, attained a lower nadir SCr, and developed acute rejection later compared to adherent patients. Almost 45% of non-adherent patients had an undetectable level of immunosuppression (calcineurin inhibitor or mammalian target of rapamycin) at presentation compared to 10% of adherent patients. A greater percentage of non-adherent patients received ATG whereas adherent patients were more likely to have received OKT3. There were no differences in eGFR, need for dialysis at presentation, Banff grade on biopsy, or presence of AMR on biopsy between the groups.

**Table 1:** Patient, Transplant and Pathologic Characteristics

	Overall *N* = 182	Adherent *N* = 111	Non-adherent *N* = 71	*p* value
Age, years	40.2 ± 13.0	42.4 ± 12.9	36.7 ± 12.5	0.003
Male	107 (59)	72 (65)	35 (49)	0.04
Race/Ethnicity				
• White	87 (48)	58 (52)	29 (41)	0.06
• Black	91 (50)	49 (44)	42 (59)	
• Hispanic	4 (2)	4 (4)	0 (0)	
Living Donor	78 (43)	50 (45)	28 (39)	0.53
Cause of ESRD^a^				
• HTN^b^	34 (19)	19 (17)	15 (21)	0.10
• DM^c^	40 (22)	31 (28)	9 (13)	
• GN^d^	59 (32)	34 (31)	25 (35)	
• PKD^e^	7 (4)	6 (5)	1 (1)	
• Other	29 (16)	14 (13)	15 (21)	
• Unknown	13 (7)	7 (6)	6 (9)	
Maintenance IS^f^				
• FK^g^/MMF^h^/Pred	101 (55)	60 (54)	41 (58)	0.12
• FK/MMF	31 (17)	14 (13)	17 (24)	
• CsA^i^/MMF/Pred	22 (12)	17 (15)	5 (7)	
• MToR^j^-based	14 (8)	11 (10)	3 (4)	
• Other	14 (8)	9 (8)	5 (7)	
Steroid withdrawal	33 (18)	13 (12)	20 (28)	0.005
Nadir baseline SCr^k^, mg/dL	1.52 ± 1.19	1.73 ± 1.45	1.19 ± 0.45	0.002
Index eGFR^l^, mL/min/1.73m^2^				
• > 30	34 (19)	24 (22)	10 (14)	0.13
• 11–30	91 (50)	58 (52)	33 (46)	
• ≤ 10	57 (31)	29 (26)	28 (40)	
Need for dialysis on presentation	34 (19)	18 (16)	16 (22)	0.29
Undetectable IS	43 (24)	11 (10)	32 (45)	< 0.001
DSA^m^ identified	59 (36)	29 (30)	30 (47)	0.02
Time from last follow-up to rejection, days				
• Mean ± SD	107 ± 322	58 ± 149	184 ± 476	< 0.001
• Median (IQR)	42 (10–103)	21 (6–60)	91 (47–140)	
Time from transplant to rejection, days				
• Mean ± SD	1140 ± 3091	1052 ± 3809	1279 ± 1368	< 0.001
• Median (IQR)	396 (103–1251)	193 (28–953)	827 (327–1829)	
Rejection <30d from transplant	31 (17)	28 (25)	3 (4)	< 0.001
Lymphocyte-depleting agent				
• Rabbit ATG^n^	125 (68)	66 (59)	58 (82)	0.009
• Muromonab	48 (26)	39 (35)	9 (13)	
• Alemtuzumab	7 (4)	4 (4)	3 (4)	
• Horse ATG	3 (2)	2 (2)	1 (1)	
Banff Grade				
• Banff I (A + B)	90 (50)	50 (45)	40 (56)	0.18
• Banff II (A + B)	77 (42)	53 (48)	24 (34)	
• Banff III	15 (8)	8 (7)	7 (10)	
AMR^o^	57 (31)	30 (27)	27 (38)	0.12
Percent Interstitial fibrosis				
• < 5%	99 (55)	29 (26)	25 (35)	0.005
• 5–25%	46 (25)	64 (58)	24 (34)	
• > 25%	37 (20)	18 (16)	22 (31)	
Percent Glomerulosclerosis				
• 0	99 (55)	68 (61)	31 (43)	0.02
• 1–20%	46 (25)	27 (24)	19 (27)	
• > 20%	37 (20)	16 (15)	21 (30)	
Plasma cell or eosinophil-rich	51 (28)	24 (22)	27 (38)	0.02
Transplant glomerulopathy	51 (28)	22 (20)	29 (41)	0.003
C4d positivity	58 (32)	30 (27)	28 (39)	0.08

Continuous variables are presented as mean (standard deviation) and categorical variables as N (%) except when noted otherwise

^a^end-stage renal disease; ^b^hypertension; ^c^diabetes mellitus; ^d^glomerulonephritis; ^e^polycystic kidney disease; ^f^immunosuppression; ^g^tacrolimus; ^h^mycophenolate mofetil; ^i^cyclosporine; ^j^mammalian target of rapamycin; ^k^serum creatinine; ^l^estimated glomerular filtration rate; ^m^donor specific antibody; ^n^anti-thymocyte globulin; ^o^antibody-mediated rejection

**Fig. 1:**
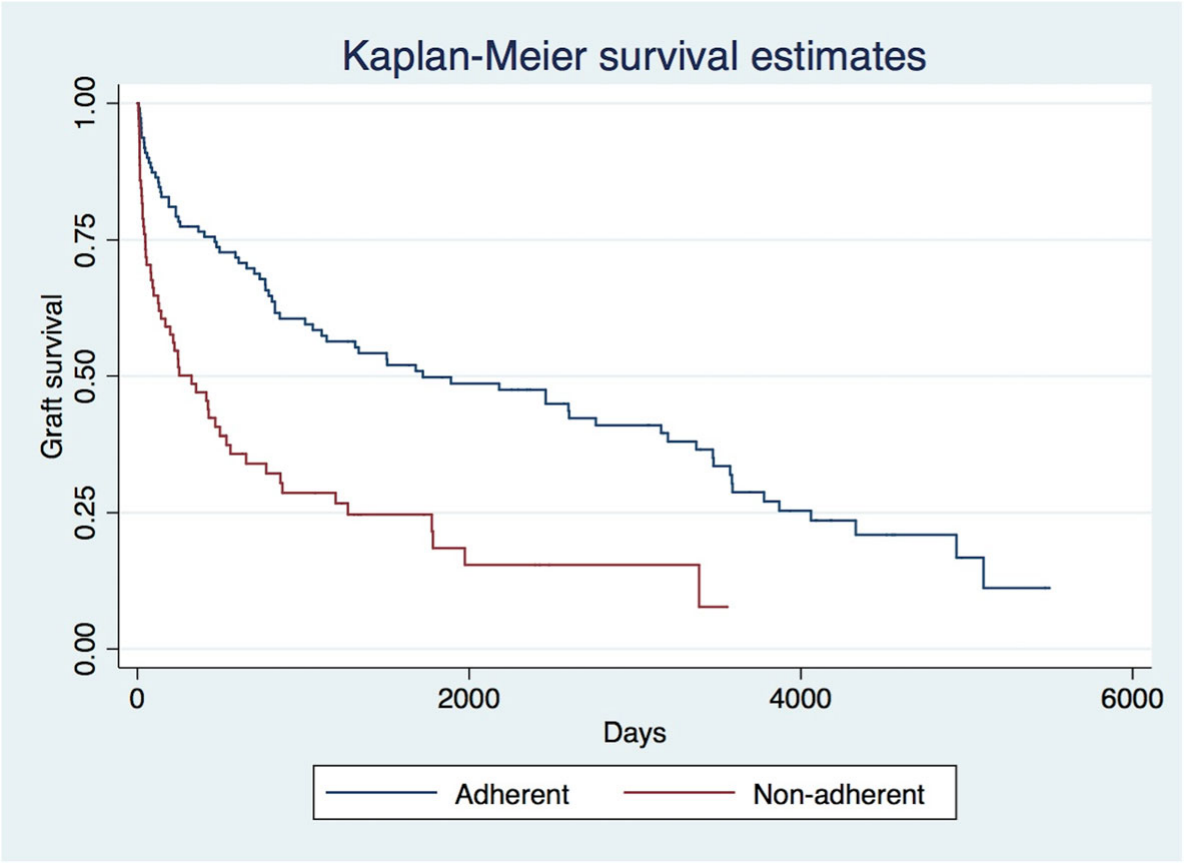
Kaplan-Meier Graft Survival Estimates After Acute Rejection Treatment

Overall, 48 (26%) patients lost their grafts within 6 months of acute rejection treatment, of which 2 were deaths. At 12 months after acute rejection treatment, 62 (34%) patients lost their grafts. The non-adherent group had more graft losses within 6 and 12 months of acute rejection treatment compared to the adherent group (41% vs 17%, *p* < 0.001; 52% vs 23%, *p* < 0.001, respectively). Time to all-cause graft loss after acute rejection treatment was significantly shorter in the non-adherent group [median 142 days (IQR 31–536)] versus the adherent group [median 781 days (IQR 144–2462)] (Fig. 1, log rank test *p* < 0.001).

In multivariable logistic regression analysis, non-adherence was associated with all-cause graft loss at 6 months after acute rejection treatment [OR 2.64 (95% CI 1.23–5.65), *p* = 0.012] after adjusting for eGFR on presentation, Banff grade, presence of AMR, and degree of interstitial fibrosis **(**Table 2**)**. When examining the secondary outcome of graft loss at 12 months **(**Table 3**)**, the results were similar [OR 3.24 (95% CI 1.58–6.66), *p* = 0.001]. An eGFR of less than 15 mL/min/1.73m^2^ on presentation and the presence of AMR were also associated with graft loss at both 6 and 12 months. The degree of interstitial fibrosis was significantly associated with graft loss at 6 months but not at 12 months.

**Table 2:** Multivariable Logistic Regression Analysis for All-Cause Graft Loss by 6 months

All-cause graft loss < 6 months	Odds Ratio (95% Confidence Interval)	*P* value
Non-adherence (ref: adherence)	2.64 (1.23–5.65)	0.01
eGFR^a^ < 15 at presentation (ref: eGFR > 15)	4.71 (2.09–10.61)	< 0.001
Banff grades II or III (ref: Banff grade I)	0.95 (0.44–2.02)	0.88
AMR^b^ (ref: no AMR)	2.46 (1.14–5.32)	0.02
Interstitial fibrosis (per 1% increase)	1.02 (1.00–1.04)	0.03

^a^estimated glomerular filtration rate (mL/min/1.73m^2^); ^b^antibody mediated rejection

**Table 3:** Multivariable Logistic Regression Analysis for All-Cause Graft Loss by 12 months

All-cause graft loss < 12 months	Odds Ratio (95% Confidence Interval)	*P* value
Non-adherence (ref: adherence)	3.24 (1.58–6.68)	0.001
eGFR^a^ < 15 at presentation (ref: > 15)	4.57 (2.19–9.53)	< 0.001
Banff grades II or III (ref: Banff grade I)	0.79 (0.39–1.62)	0.53
AMR^b^ (ref: no AMR)	2.71 (1.30–5.68)	0.01
Interstitial fibrosis (per 1% increase)	1.01 (0.99–1.03)	0.31

^a^estimated glomerular filtration rate (mL/min/1.73m^2^); ^b^antibody mediated rejection

In the Cox proportional hazards model **(**Additional file 1: Table S1**)**, non-adherence was associated with an increased risk of all-cause graft loss over time (HR 1.81, 95% CI 1.20–2.73), after adjustment for age at rejection, race, type of transplant, nadir SCr, eGFR at presentation for rejection, Banff grade, presence of AMR, degree of interstitial fibrosis and lymphocyte depleting agent used.

In sensitivity analysis, results of the modified poisson regression with robust variance model were consistent with the logistic regression model. Non-adherence was significantly associated with all-cause graft loss at 6 months after acute rejection treatment [RR 1.83 (95% CI 1.12–2.98), *p* = 0.016], after adjusting for eGFR on presentation, Banff grade, presence of AMR, and degree of interstitial fibrosis **(**Additional file 2: Table S2).

## Discussion

In this study, we found that patients who were determined by their clinical team to be non-adherent with their immunosuppression were significantly more likely to lose their allografts within 6 and 12 months of a severe acute rejection episode, despite treatment with a T-lymphocyte depleting agent. This association was independent of the eGFR on presentation, presence of AMR, Banff grade and degree of interstitial fibrosis. Notably, there were no differences in eGFR on presentation, distribution of Banff grade or presence of AMR when comparing adherent versus non-adherent patients. Other identified risk factors for short-term allograft loss after severe acute rejection treatment were an eGFR of < 15 mL/min/1.73m^2^ on presentation, presence of AMR and a higher degree of interstitial fibrosis.

Identifying patients who are at high risk for short-term allograft loss despite treatment is important in individualizing clinical decision making. If allograft survival is likely to be limited to only a few months despite potent treatment, the clinician may choose to acknowledge the likely loss of the allograft and withhold administration of agents such as ATG that carry significant risks. The focus of the therapeutic plan should instead perhaps shift towards ESRD planning.

Prior studies have shown that various histological markers are indicative of a higher risk of allograft loss following acute rejection. For example, Banff grade III, and tubulitis and interstitial inflammation in the setting of vascular involvement, correlated with a higher incidence of irreversible graft loss, which was assessed by the SCr response at 2 weeks following treatment for rejection [14]. It has also been demonstrated that eGFR at diagnosis of acute rejection and density of plasma cell infiltration are associated with return to dialysis [18]. In our study, we similarly found eGFR to be an important predictor of allograft loss after acute rejection but did not find Banff grade to be a significant factor.

To our knowledge, no prior studies have specifically focused on examining the relationship of acute rejection and short-term allograft loss in the setting of non-adherence. A study by Morrissey et al. [19] found no difference in graft survival if the rejection was secondary to non-adherence, although the authors did not study short-term allograft loss as an outcome. Others have shown that non-adherence results in acute rejection and eventual graft loss [20]. Self-reported non-adherence, immunosuppressant trough variability and percentage of sub-therapeutic trough levels have also been separately correlated with late allograft rejection [21].

Our findings suggest that non-adherence is an independent risk factor for short-term allograft loss after an episode of severe acute rejection despite aggressive treatment. One potential mechanism that could explain this association is the nature of pathologic injury and resultant histological changes that we hypothesize could make patients more resistant to standard treatments. Non-adherence has been previously associated with acute rejection at one-year post transplant as well as poor clinical outcomes at 5 years following transplant, and the biopsies of this patient population identified a histological variant of late acute rejection associated with non-adherence [16]. Notably, patients who were non-adherent with their immunosuppression demonstrated a higher percentage of acute cellular rejection based on Banff criteria, a higher percentage of tubulitis, less interstitial edema and more interstitial inflammation. In addition, the relatively small study found that patients who were deemed severely non-adherent, based on higher variability of cyclosporine levels, were more likely to have a denser inflammatory infiltrate. In comparison, our study identified several significant differences in pathological characteristics wherein non-adherent patients had more interstitial fibrosis and glomerulosclerosis, and a greater percentage developed plasma cell or eosinophil-rich rejections, DSA and transplant glomerulopathy. However, the distribution of Banff grade or concomitant AMR were not significantly different between the two groups. These findings warrant further investigation focusing on histological differences based on adherence and outcomes potentially associated with these findings. Although this study only examined associations and not causal relationships, we hypothesize that the presence of plasma cell or eosinophil-rich rejection, and development of DSA and transplant glomerulopathy are on the causal pathway for why non-adherence with severe acute rejection is more likely to lead to early graft loss.

Another potential mechanism explaining our findings is that patients who are non-adherent to their immunosuppression, remain non-adherent after they receive acute rejection therapy. This pattern of behavior would attenuate the beneficial response that they could have achieved with therapy. This patient population may benefit from closer follow-up after treatment of acute rejection for monitoring of medication adherence.

The strength of this study is that it addressed a common and important clinical question utilizing a clinically relevant and practical outcome. The study’s findings can help guide clinical decision making by identifying patients at increased risk for poor short-term outcomes. This information may also be helpful to improving shared decision making and informing patients of their prognosis. Additionally, this study highlights the negative influence of non-adherence in allograft outcomes and underscores the importance of considering this issue prior to initiating aggressive treatments.

This study has several limitations. First, the classification of adherence status was clinically adjudicated by the provider caring for the patient and his or her subsequent documentation in the medical record. This criterion is subjective and susceptible to misclassification bias. However, our findings apply to the usual scenario by which non-adherence is determined by kidney transplant clinicians. Further, non-differential misclassification bias would be expected to bias the study to the null. Future studies should further delineate clinical non-adherence to determine if particular subsets of clinically non-adherent patients (e.g. self-report, undetectable immunosuppressive levels) are at increased risk of early graft loss. Second, this was a single-center study and our findings need to be duplicated elsewhere to ensure generalizability to patients treated at other centers that may have different acute rejection treatment protocols. Third, due to the observational nature of the study and the relatively small number of events limiting regression model size, residual confounding may exist. We were unable to include other potential confounders in the multivariable regression analysis such as presence of plasma cell or eosinophil-rich rejection, DSA, transplant glomerulopathy, or being on a steroid withdrawal protocol. In the future, as we accumulate more cases, we believe it will be worthwhile to repeat the analyses to include these variables.

## Conclusions

In conclusion, patients who are non-adherent are at increased risk for short-term allograft loss after a severe acute rejection despite T-lymphocyte depleting therapy. Other significant predictors include severe renal dysfunction on presentation, presence of AMR and a greater degree of interstitial fibrosis. These findings may aid clinicians in risk stratifying patients for poor short-term outcomes and treatment futility.

## Supplementary information

**Additional file 1: Table S1.** Cox Proportional Hazards Model for All-Cause Graft Loss.

**Additional file 2: Table S2.** Modified Poisson Regression with Robust Variance Analysis for All-Cause Graft Loss by 6 Months.

## Data Availability

The dataset used for the study is available without patient identifiers from the corresponding author on reasonable request.

## Abbreviations

AMR: Antibody-mediated rejection
ATG: Anti-thymocyte globulin
CCTT: Cooperative Clinical Trials in Transplantation
DSA: Donor specific antibody
eGFR: Estimated glomerular filtration rate
ESRD: End-stage renal disease
OKT3: Muromonab-CD3
SCr: Serum creatinine
