# Supplementary material for: Association of medication non-adherence with short-term allograft loss after the treatment of severe acute kidney transplant rejection
Source: BMC Nephrol. 2019 Oct 17;20:373. doi: 10.1186/s12882-019-1563-z (PMC6796330; doi:10.1186/s12882-019-1563-z)
Supplement: Supplementary file 1 — Additional file 1: Table S1. Cox Proportional Hazards Model for All-Cause Graft Loss. [file 12882_2019_1563_MOESM1_ESM.docx]

**Table S1.** Cox Proportional Hazards Model for All-Cause Graft Loss

| **All-cause graft loss** | **Hazard Ratio (95% Confidence Interval)** | **P value** |
| --- | --- | --- |
| Non-adherence (ref: adherence) | 1.81 (1.20-2.74) | 0.005 |
| eGFR^a^ <15 at presentation (ref: >15) | 2.23 (1.53-3.25) | <0.001 |
| Banff grades II or III (ref: Banff grade I) | 0.81 (0.56-1.18) | 0.28 |
| AMR^b^ (ref: no AMR) | 1.60 (1.07-2.37) | 0.02 |
| Interstitial fibrosis (per 1% increase) | 1.02 (1.01-1.03) | <0.001 |
| Age at rejection (per 1 year increase) | 0.99 (0.08-1.01) | 0.38 |
| Non-white race (ref: white) | 1.59 (1.12-2.24) | 0.01 |
| Deceased donor transplant (ref: living) | 1.10 (0.74-1.62) | 0.63 |
| Nadir SCr^c^ (per 1 mg/dL increase) | 0.78 (0.62-0.98) | 0.04 |
| Non-ATG^d^ treatment (ref: ATG) | 0.93 (0.61-1.41) | 0.72 |

^a^estimated glomerular filtration rate (mL/min/1.73m^2^); ^b^antibody mediated rejection; ^c^serum creatinine; ^d^anti-thymocyte globulin
