# Supplementary material for: Association of medication non-adherence with short-term allograft loss after the treatment of severe acute kidney transplant rejection
Source: BMC Nephrol. 2019 Oct 17;20:373. doi: 10.1186/s12882-019-1563-z (PMC6796330; doi:10.1186/s12882-019-1563-z)
Supplement: Supplementary file 2 — Additional file 2: Table S2. Modified Poisson Regression with Robust Variance Analysis for All-Cause Graft Loss by 6 Months. [file 12882_2019_1563_MOESM2_ESM.docx]

**Table S2.** Modified Poisson Regression with Robust Variance Analysis for All-Cause Graft Loss by 6 Months

| **All-cause graft loss <6 month** | **Relative Risk (95% Confidence Interval)** | **P value** |
| --- | --- | --- |
| Non-adherence (ref: adherence) | 1.83 (1.12-2.98) | 0.016 |
| eGFR^a^ <15 at presentation (ref: >15) | 2.80 (1.57-4.98) | <0.001 |
| Banff grades II or III (ref: Banff grade I) | 0.89 (0.56-1.42) | 0.62 |
| AMR^b^ (ref: no AMR) | 1.72 (1.09-2.71) | 0.02 |
| Interstitial fibrosis (per 1% increase) | 1.01 (1.00-1.02) | 0.003 |

^a^estimated glomerular filtration rate (mL/min/1.73m^2^); ^b^antibody mediated rejection
